# Acquired Brain Injury and Interventions in the Offender Population: A Systematic Review

**DOI:** 10.3389/fpsyt.2021.658328

**Published:** 2021-05-07

**Authors:** Esther Q. J. de Geus, Maarten V. Milders, Joan E. van Horn, Frank A. Jonker, Thijs Fassaert, Juliette C. Hutten, Femke Kuipers, Christel Grimbergen, Siri D. S. Noordermeer

**Affiliations:** ^1^Department of Clinical Neuro- and Development Psychology, Faculty of Behavioral Sciences, Section of Clinical Neuropsychology, Vrije Universiteit Amsterdam, Amsterdam, Netherlands; ^2^De Waag, Amsterdam, Netherlands; ^3^Altrecht, Vesalius, Amsterdam, Netherlands; ^4^Public Health Services, Amsterdam, Netherlands

**Keywords:** forensic, prison, intervention, recidivism, acquired brain injured, offenders

## Abstract

**Background and Aims:** The prevalence of acquired brain injury (ABI) in offender populations appears much higher than in the general population, being estimated at 50% compared to 12%, respectively. Taking into account ABI-related cognitive and social impairments or behavioral changes in forensic treatments might be relevant and may improve treatment outcomes. The aim of the current review is to summarize and integrate the literature on psychological interventions or treatments for consequences of ABI in the forensic setting. Reviewing this literature could provide crucial information for improving treatment options for offenders with ABI, which may contribute to reducing recidivism.

**Methods:** The PubMed/MEDLINE, PsychInfo, CINAHL, COCHRANE, and Web of Science databases were searched for studies in adult offenders with ABI that evaluated the effect of psychological interventions with a focus on ABI-related impairments and recidivism.

**Results:** This review identified four intervention studies that met the inclusion criteria. These included an adult population (≥18-year-old) in a forensic setting (given the focus of the current review on treatment, defined here as an environment in which offenders are treated while being incarcerated or as outpatients), non-pharmacological treatments and were published in English or Dutch between 2005 and 2020. All studies reported some positive effects of the intervention on interpersonal behavior, cognition and recidivism. The aspects of the interventions that seemed most beneficial included personalized treatment and re-entry plans, support for the individual and their environment and psychoeducation about the effects of ABI.

**Discussion:** Although positive effects were reported in the studies reviewed, all studies had methodological limitations in terms of sample size, study design and outcome measures which affects the strength of the evidence. This limits strong conclusions and generalizability to the entire offender population.

**Conclusion:** Despite high prevalence of ABI in offender populations, interventions in forensic settings seldom address the effect of ABI. The few studies that did take ABI into account reported positive effects, but those results should be interpreted with caution. Future studies are warranted, since this does seem an important venue to improve treatment, which could eventually contribute to reducing recidivism.

## Introduction

Several studies in offender populations, which includes prisoners and in-patients and out-patients in forensic psychiatric settings, have reported considerably high acquired brain injury (ABI) prevalence estimates, with a mean estimated at 50%, ranging from 6% to 100% (1–4). This finding is important, as ABI-related cognitive and social impairments are associated with several deficits, among which are behavioral deficits such as aggression, substance abuse and even criminal behavior (2, 5). Research showed that ABI-related cognitive and social impairments contributed to more (previous) convictions and higher recidivism rates (6, 7). This underlines the importance of ABI awareness in forensic settings (i.e., environments in which offenders are treated while being incarcerated or as outpatients), and offender interventions, to possibly improve treatment options and ultimately reduce recidivism.

ABI is defined as “an injury to the brain that is not congenital, degenerative, hereditary or caused by a birth trauma” (8), and can be the result of both traumatic and non-traumatic causes. Non-traumatic causes include stroke, infection, tumor, or oxygen deficiency, affecting the brain. Traumatic brain injury (TBI) occurs when an external force injures the brain, with or without penetration of the skull, such as with falls, traffic accidents, or violence (9). Negative outcomes are seen in cognitive, emotional, and behavioral domains following all ABI types, but especially following TBI types (10, 11). The neurocognitive and behavioral consequences can be extensive and disabling. There is evidence that cognitive behavioral therapy (CGT), behavioral management techniques and metacognitive strategy training (e.g., self-monitoring, self-regulation and time pressure management) are effective interventions for ABI outcomes, such as aggression, executive dysfunction and social communication problems. For memory impairments it is recommended to use internalized strategies and external memory compensations. For all interventions it is important to promote generalization to daily functioning (12, 13).

Prevalence estimates of ABI in the general adult population vary widely, where prevalence rates between 1 and 35% have been reported (14–18). More specifically, the average prevalence of TBI is estimated at 12% according to a meta-analysis (19), whereas the prevalence of non-traumatic brain injury (stroke) is estimated at 5–10% (20). Variations in reported prevalence rates are largely due to the use of different definitions, assessment methods, and samples included. For instance, high prevalence rate studies included information from all ABI types, regardless of severity, collected through self-report, whereas low prevalence rate studies often only included cases based on objective TBI measurements from hospitalized TBI survivors (17). In comparison with the general population, prevalence of ABI in the offender population is considerably higher (21), and those estimations are likely to be underestimations because research in offender populations typically only focuses on TBI, and information about non-traumatic brain injury is usually not available. With regard to a specific subgroup of the offender population, namely forensic psychiatric patients who undergo treatment, comparable high prevalence rates have been reported (21, 22). The prevalence and extent of brain pathology in institutionalized offenders, compared to non-offenders, was significantly higher with a prevalence of 46% vs. 8% (21).

As in the general population, different definitions, assessment methods, and sample populations contributed to the wide variations in prevalence rates. More specifically, characteristics of the sample population that can contribute to higher TBI prevalence rates include low socioeconomic status and sex, with men being up to twice as likely to suffer a TBI than women (15, 19, 23). Age is also a contributing factor to the prevalence estimates, where men between the age of 18 and 25 are at a relatively high risk of TBI due to risk-taking behaviors, and individuals over 70 are at a higher risk of TBI due to falls (14, 19). Furthermore, different assessment methods of TBI have been used in forensic settings. In-depth interviews conducted by a trained psychological professional resulted in more accurate assessments and higher prevalence rates of TBI than the more frequently used short screening tools (1). Self-report measurements for TBI can be difficult because of memory deficits and lack of comprehension or understanding of the injury. Only a few studies used valid and reliable measurements to assess TBI, but even the use of valid and reliable screening tools does not fully account for the wide range of prevalence rates (3, 4). Lastly, different definitions of TBI are used (1). For example, taking into account the severity of TBI, often measured with the Glasgow Coma Scale (GCS), TBI can be classified as mild, moderate, or severe. Mild TBI corresponds with a high GCS score, while severe TBI corresponds with a low GCS score (24). However, this specific classification, that includes important characteristics using the GCS score, is often only reported for hospitalized TBIs, since it is difficult to obtain retroactively or with self-report (1).

The finding that TBI, as a form of ABI, is clearly more common in the offender than in the general population (25) is important, as TBI is associated with several deficits and negative outcomes. In terms of TBI-related cognitive impairments; executive functioning deficits, memory and attention deficits, and slowed information-processing are frequently reported (26–28). Negative outcomes following TBI are also seen in emotional functioning, including deficits in social communication, social cognition, emotion recognition, empathy, self-regulation or self-control and self-awareness (26, 29–31). These deficits can be related to failures to understand others, to make appropriate emotional contributions, and problems with controlling one's behavior (32, 33). TBI-related impairments in social and cognitive functions can contribute to a wide range of anti-social behaviors, such as aggression, rule-breaking, and other risk-increasing behaviors such as substance abuse, which makes TBI a risk factor for prosecution and imprisonment (2, 3, 29, 34, 35). Prevalence rates of verbal and physical aggression were reported in the range from 4% up to 88% in TBI survivors and reflected a higher risk of convictions and (re)offending (6, 7, 36, 37). Severity of TBI could further exacerbate these behavioral problems (5, 38). Several longitudinal studies confirmed the positive relationship between severity of TBI and criminal behavior and an elevated risk of developing mental disorders, such as drug and alcohol dependence (5, 39, 40).

In sum, cognitive, emotional, and behavioral changes are common outcomes of ABI. As the prevalence of ABI, which mainly consists of TBI in the offender population, appears to be high, it is likely that the associated impairments are present in a substantial proportion of the offender population. In addition to difficulties in daily life, these impairments can also have a negative impact on treatment outcomes. For example, impaired self-awareness can cause difficulties in understanding the need for treatment and has been linked to poor treatment adherence (41). Furthermore, executive dysfunctions can be misunderstood as deliberate problem behavior, which can contribute to misconduct in prison. A mismatch between treatment and capacities can lead to low treatment adherence or discontinuation of treatment. The relationship between brain injury, recidivism, and prior incarcerations has been confirmed, showing that TBI-related violence and aggression contributed to more (previous) convictions and higher recidivism rates (6, 7, 36).

The primary aim of forensic interventions is to reduce recidivism, and these are often based on the Risk-Needs-Responsivity Model (42). To date, forensic treatment, which is mostly based on cognitive-behavioral techniques, results in modest improvements in terms of recidivism reduction in only 8–30% of those who complete treatment (43–45). However, attrition rates are relatively high, on average 30% (46–48). It is possible that the modest effectiveness of forensic treatment is in part due to ABI-related impairments not sufficiently being taken into account in treatment. Treatment in forensic settings is typically developed for patients with intact cognitive functions. Thus, even though ABI-related cognitive, emotional, and behavioral impairments are presumably common in this population, it is unclear whether forensic treatment takes these impairments into account. Suggestions for improving treatment or rehabilitation programs, by improving executive cognitive functioning have been reported and include individualized assessment of deficits and individualized functional rehabilitation (49). Reducing ABI-related cognitive, emotional, and behavioral impairments could result in better treatment outcomes (both adherence and continuation) and reduced recidivism, and may therefore become important treatment goals. The aim of the current study was to review the literature on interventions and treatment in offender populations suffering from ABI, or the influence of ABI on treatment. In the long run, improved treatment options and treatment outcomes may reduce the ABI-related impairments in offenders and, ultimately reduce recidivism.

## Methods

### Search Strategy

A PRISMA systematic literature search was conducted using the following scientific databases: PubMed/MEDLINE, PsychInfo, CINAHL, COCHRANE, and Web of Science. Inclusion criteria were; research in adult populations (≥18-year-old) in a forensic setting (i.e., an environment in which offenders are treated while being incarcerated or as outpatients), non-pharmacological treatments, published in English or Dutch between 2005 and 2020. Key terms were adapted for each database and included variations of “acquired brain injury,” “traumatic brain injury,” “brain injury” OR “head injury” AND “rehabilitation,” “treatment,” “intervention,” “therapy,” “neurorehabilitation,” “management” OR “psychotherapy” AND “forensic setting,” “forensic population,” “incarcerated,” “prison” OR “offender population.” To ensure that no articles were missed in the original search, the reference list of the articles meeting the inclusion criteria were also scanned.

### Study Selection

In total 378 articles were retrieved from the databases and another 15 articles were retrieved from reference lists. After excluding duplicates, 234 articles remained. Two assessors independently conducted all the steps for article inclusion based on the inclusion criteria. Disagreements between the assessors were discussed until agreement was reached. Interrater reliability was excellent with high kappa scores for title inclusion (0.85), abstract inclusion (0.84), and article inclusion (1.00).

**Figure 1:**
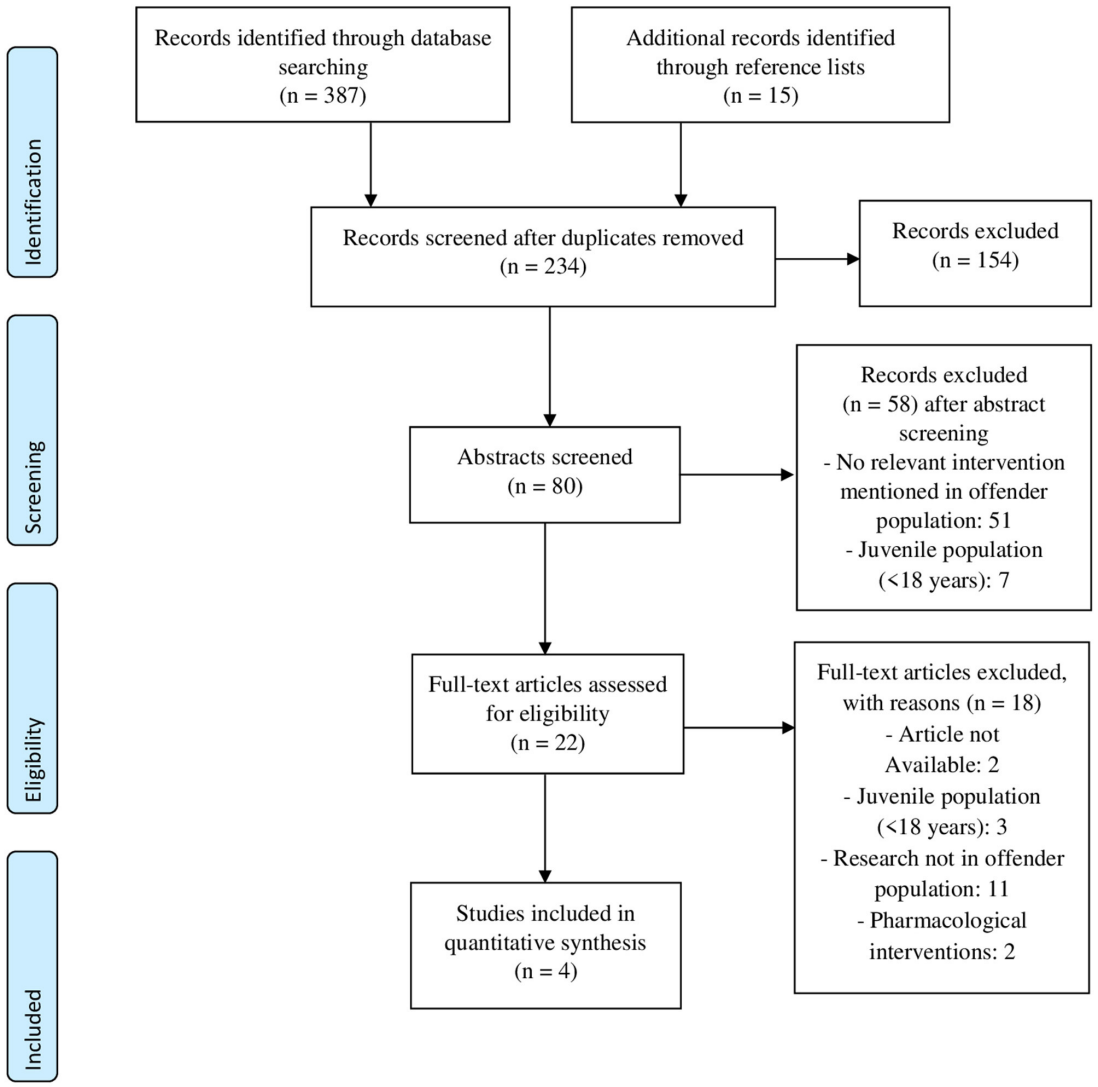
PRISMA (50) flow diagram of decision process for included studies.

After screening of the 234 article titles, 154 articles were excluded based on the title, which indicated non-compliance with the inclusion criteria of this review. The abstracts of the remaining 80 articles were screened. Based on the abstracts, 58 articles were excluded because the article failed to meet the inclusion criteria, mostly because the study population was too young (aged <18), or relevant information about the intervention was missing. The 22 remaining articles were selected and reviewed in their entirety for inclusion. Finally, four articles were identified as meeting the criteria for inclusion in this review, see Figure 1 for the PRISMA flow chart. Two of the studies were single case experimental design studies (51, 52) and two were single group experimental design studies (53, 54).

## Results

The two case studies had a total of 6 participants (all male) (51, 52) and the two group studies had a total of 80 participants (3 female and 77 male) (53, 54). In all studies, participants received a type of psychological intervention or treatment for consequences of ABI in the forensic setting. The characteristics of each study, including age, gender, sample size, intervention, outcome measure, setting of the intervention, and country are summarized in Table 1.

**Table 1:** Characteristics of studies included within the current review.

**Autor/Year**	**Design**	**N (pre)**	**N (post)**	**Follow-up**	**Age (years)**	**Gender**	**Time of first TBI**	**TBI assessment**	**Measured outcomes**	**Summary intervention**	**Findings**	**Setting (country)**
Manchester, Wall, Dawson & Jackson (2007)	Single case design	3	2	3 months	19 19 21	3 M	12 years 17 years 19 years	Medical records (CGS and PTA).	“How I Think Questionnaire” (HIT), Coopersmith Self-Esteem Inventory (SEI), Overt Aggression Scale (OAS).	Equip intervention program for improving social interaction skills, sociomoral development and social cognitive distortions.	Moderate effects were found for HIT outcomes, pro-aggressive attitudes and beliefs were modified in two patients, all participants showed reductions of aggression (OAS), and little effect on self-esteem was found (SEI).	Rehabilitation facility(United Kingdom; U.K.).
Ramos, Oddy, Liddement & Fortescue (2018)	Single case design	3	3	N/A	22 47 40	3 M	2 years 25 years 10 years	Brain Injury Screening Index (BISI) and medical records.	Independence, Constructive activity, reoffending/prison infractions, use of cognitive strategies.	Link worker intervention.	Link worker intervention can lead to positive outcomes and that help with identifying and intervening problems seems to be feasible.	Prison (U.K.).
Marcer, Mills & Clarke (2016)	Single group design	13	13	N/A	Mean 33.9	10 M 3 F	N/A	Demo-graphic and clinical information.	Different executive functions with repeated measures design.	Cognitive remediation therapy (CRT).	CRT demonstrates improvement in cognitive functioning.	Low secured prison, mental health department (U.K.).
Nagele, Vaccaro, Schmidt & Keating (2018)	Single group design	67	44	2 years	N/A	67 M	75% occurred in child-hood (<21 years)	Traumatic Brain Injury Question-naire (TBIQ).	Employment, re-incarceration, violation of parole.	Intervention program named NeuroResource Facilitation (NRF) with goal to reduce recidivism and improve productivity.	The intervention program showed a reduction in recidivism and an increase in productivity.	Maximum secured prison (United States of America; U.S.A.).

### Equip: A Forensic Peer Group Approach

The multiple case study by Manchester et al. (51) included Equip, a forensic peer group approach for young adults (18–23 years), to study bullying behavior, aggression, and antisocial attitudes after TBI in a rehabilitation facility. All three participants had sustained severe TBI and had a history of criminality. The participants were highly resistant to other forms of treatment, such as neurobehavioral rehabilitation. The Equip intervention program focused on social interaction skills, sociomoral development, and social cognitive distortions, such as moral misjudgments, aggressive and impulsive reactions, and egocentric biases. The program consisted of four 30-min group sessions per week, for 6 weeks. Outcome measures were two self-report questionnaires, the How I Think Questionnaire (HIT), measuring self-serving cognitive distortions with externalizing pathology, and the Coopersmith Self-Esteem Inventory (SEI), measuring evaluative self-attitudes, completed by the participants before and after the program. Aggressive behaviors were recorded by the staff with the Overt Aggression Scale (OAS), 2 weeks prior to the start of the intervention program, 2 weeks after the end of the program, and 2 weeks after the three-month follow-up. After completing the program, two of the three participants had altered their beliefs regarding antisocial behavior, for example, a reduction in pro-aggressive beliefs was seen. At the three-month follow-up, one participant maintained this progress, while the others returned to baseline. A reduction in verbal aggression was seen in all participants after completing the program. Therefore, the authors suggested that a group approach may help modify underlying antisocial behaviors and attitudes and verbal aggression in patients with severe TBI. However, the causal relationship cannot be determined due to the absence of multiple baseline measurements and of a control group. Furthermore, the very small sample size and the heterogeneity of the sample (all participants had different intervals between brain injury, admission to a rehabilitation facility, and start of the group program) were limitations of this study. Lastly, it was unclear how treatment effects could be maintained once a client had left the structured rehabilitation environment (51).

### Cognitive Remediation Therapy (CRT)

The second study, by Marcer et al. (53), found improvements in cognitive functions following cognitive remediation therapy (CRT) in a sample of offenders with complex mental health problems (e.g., personality disorder) in addition to TBI and/or substance abuse. The single group design study consisted of a small sample (*N* = 13) with only two participants suffering from TBI. The remaining 11 participants were diagnosed with substance abuse and personality disorders, without TBI (*N* = 6) or of whom it was unknown whether they had suffered from TBI (*N* = 5). CRT is a cognitive and behavioral manualized intervention that aims to improve cognitive abilities. With drill and practice techniques, strategy implementation, and application of principles of learning such as errorless learning and scaffolding, CRT aims to improve cognitive abilities. The participants started with a 2-weekly half-hour introduction (1-h total), where they increased their understanding of cognitive skills and enhanced their engagement in CRT. After the introduction the participants completed a full battery of cognitive tests, the pre-intervention assessment. In the 14-week full program the participants received five CRT modules which each consisted of 8 sessions (40 total) and completed approximately 15 tasks per module (total of 75 tasks). During those sessions the participants had to think about the most helpful strategy to solve a cognitive task, reflect on strategies, and evaluate the effectiveness of the strategy in improving their performance. Inhibition, rule shifting, planning and problem solving, and attention and working memory were assessed pre- and post-intervention. Participants' performance in terms of inhibition, rule shifting, attention, and working memory, including performance of participants with TBI, had improved post-intervention. In addition, the authors performed a sensitivity analyses comparing the patients with TBI (*N* = 2) and those without TBI (*N* = 6), showing equal gains from the CRT. Therefore, the authors concluded that CRT improved cognitive functioning in all patients, including those with TBI. However, there were several limitations to this study. First, the sample was small, especially the TBI group, and no control group was included. Second, because the same tasks were used at pre- and post-intervention, rather than parallel versions of the tasks, it is possible that improvements were produced by practice effects. Finally, long-term effects of the CRT intervention were not considered.

### Link Worker or Facilitator Intervention

Ramos et al. reported a link worker intervention offered to three prison inmates who reported severe TBI or multiple mild TBIs (52). Link workers were usually psychology graduates who received a training about TBI, including psychoeducation on the causes and consequences of TBI, coping with the impact of TBI, and how to address problems. This service approach was designed to identify and support inmates with TBI. The link worker could respond to specific needs and their role compromised support, guidance, and providing psychoeducation to both staff and inmate about TBI. In addition, link workers tried to set up a support system for after the inmate's release. The link worker helped to make a support plan, formulate goals, and identify steps required to meet those goals. The intervention consisted of a 30–60 min session, 1–3 times a week, for ~8–12 weeks. One of the three participants was released during the intervention. He did not recidivate during the follow-up period of 3 years and was able to live independently with only little support. The other two participants were still in prison by the end of the study, but they showed no violations of prison rules since the intervention. One had successfully learned to apply support plans with basic guidelines to organize himself, his life and to successfully undertake the tasks he needed to do. Thereby he replaced his challenging behavior with constructive behavior. The third participant had no further infractions since the intervention. Before, he had memory difficulties and during the intervention, he learned to ask for information in small chunks so that he could understand and remember information better. Furthermore, he gained the “advisor role” qualification with charity work in prison after the intervention and his role was advising inmates close to release. These three case studies suggest that a link worker can lead to positive outcomes and that helping inmates in identifying and intervening with problems may be feasible. Limitations of the study were the small sample, the absence of a control group, and no multiple long-term follow-ups. Furthermore, no standardized treatment protocols or guidelines where used, which makes it difficult to repeat this intervention. It remains unclear whether the positive outcomes could be fully attributed to the intervention and whether these effects persisted over time.

Nagele et al. employed a model of TBI screening for men in a maximum secured prison (54). Of the 158 inmates screened in a semi-structured interview conducted by a staff member, 75% had a history of TBI. Additionally, 74% had neurocognitive impairments based on results of a neurocognitive test battery, testing executive function and memory abilities/capacities. The researchers included 67 participants with neurocognitive impairments likely to interfere with successful re-entry into the community once released, in a 2-year intervention program, named NeuroResource Facilitation (NRF). Neurocognitive impairments investigated were (working) memory, attention, initiation, organization, problem-solving, inhibition of behavior, self-monitoring, planning/anticipation, and mental flexibility. NRF was a service designed to identify needs, resources and provide support to individuals with TBI and their families. The goal of the intervention was to reduce recidivism and improve productivity (work, volunteering or training) of the incarcerated participants. The participants received person-specific psychoeducation and help regarding identifying goals and needs, re-entry planning and resource application from a facilitator, comparable to a link worker. For example, a personalized treatment plan to prepare the individual for return to society was formulated. There was also the possibility to join an eight-week support group, that provided the possibility to talk about all sorts of TBI related problems with fellow sufferers and professionals. After release from prison (44 participants) the facilitator and the participant met approximately twice a month for 1 year to implement the personalized re-entry plan. Those meetings focused on supportive counseling, crisis management, and learning and applying strategies. Outcome measurement at 2 years after release showed that 65% of those released were engaged in some kind of productive activity and 50% had a full- or part-time job. Only 17% were re-incarcerated within 2 years due to new convictions or violation of parole, which contrasts with typical re-incarceration rates after 2 years of ~50% in the USA (55, 56). Although the findings were promising, the study also had limitations, of which the primary limitation was the use of self-report to screen for TBI and the absence of a control group. Thus, it remains unclear whether the effects can be attributed to the intervention.

## Discussion

Previous literature showed that prevalence rates of ABI in offender populations are high. The aim of this review was to provide an overview of the literature on interventions in adult offender populations with ABI. Given the relatively high prevalence rates of ABI in offender populations, interventions aimed at ABI-related impairments could have added value in reducing recidivism rates, by improving treatment outcomes. With only four studies identified, the literature search revealed a paucity of studies reporting such interventions. In addition, the reported evidence for the effectiveness of the interventions was weak, mainly due to methodological shortcomings of the studies, in particular lack of control conditions, small sample sizes and no long-term outcomes.

All four included studies reported some improvements, albeit in different outcome domains. Manchester et al. (51) focused on aggression and antisocial behavior Marcer et al. (53), on cognitive functions and Ramos et a The link workers in Ramos et al. (52) and Nagele et al. (54) on recidivism, i.e., chance of re-offending. The reported studies also differed in how presence of ABI was assessed, their approach, and focus. Therefore, providing an integral and overall conclusion is difficult, but the positive outcomes are encouraging and warrant further investigation.

The focus of the interventions ranged from strategy learning and training cognitive functions [CRT intervention; (47)], looking into improvement of antisocial attitudes and behavior by focusing on social interaction skills, sociomoral development and social cognitive distortions [the Equip program; (45)], using a facilitator or a link worker, to provide psychoeducation, guidance, help the individual to identify goals and needs and provide support with re-entry into the community [NRF; (54)– link worker; (46)]. The NRF and link worker programs intended to reduce recidivism and problem behavior and improve productivity (52, 54). Although there was a difference in strategy and focus, the focus on psychoeducation about ABI, identification of weaknesses and strengths, providing learning strategies and giving support was shared between the programs and together these comprise the clinical implications. Psychoeducation was not only given to the incarcerated individual, but also to prison staff and family members. With identification of weaknesses and strengths it was possible to formulate a personal (re-entry) plan with corresponding goals.

The primary aim of treatments in offender populations is to reduce recidivism. Two of the included studies assessed recidivism in participants (52, 54) and both reported promising results. Both studies involved link worker interventions, where personalized treatment and re-entry plans, providing support and psychoeducation about TBI contributed to reducing recidivism. These interventions strategies are in line with the Risk-Need-Responsivity model for offender rehabilitation (42). This rehabilitation model relies on three basic principles stating that forensic treatment is most effective when (1) treatment dosage is tuned to an offender's risk level (high risk offenders are to receive more intensive treatment (risk principle); (2) treatment is targeted at the offender's dynamic (i.e., changeable through intervention) risk factors most strongly associated with criminal behavior (need principle); and (3) treatment approach is tailored to individual characteristics, such as motivation and intellectual functioning (responsivity principle). Not considering possible TBI-related cognitive and social impairments could be problematic for the responsivity principle.

Following the WHO guidelines (57), mental healthcare in forensic settings should include screening for mental disorders, addressing views and needs (of different groups), provide awareness training or psychoeducation to staff members and continued care (58). Support during the months immediately following release from prison, may be helpful for all former inmates, regardless of whether they have sustained ABI. Psychological treatment in prison and forensic outpatient facilities is often based on cognitive-behavioral techniques and has moderately positive outcomes (45). The emphasis is on specialized individual treatment, provided by a qualified (neuro-) psychologists (59). However, not all forensic settings employ qualified psychologists educated in offering specialized individual treatment focused on ABI. What the studies reviewed here suggest is that interventions can be presented by staff who are not fully qualified clinicians as well. The link workers in (52) had a psychology degree, but no further professional training and used manual-based interventions, such as EQUIP. This suggests the possibility of involving a broader range of staff in presenting the interventions, as long as they are familiarized with the intervention.

At the moment, standard treatment in forensic settings does not take the presence and consequences of ABI into account. More awareness of the risk and the consequences of ABI in forensic settings and of ABI among the psychologists and other staff will hopefully result in (developing) more suitable treatments and psychoeducation on the consequences of brain injury, with a focus on impairments in cognition and social cognition.

The studies reviewed show some positive outcomes and clinical implications, however, they have methodological weaknesses and other limitations. The absence of control conditions and a matched control group prevents strong conclusions regarding the effectiveness of the interventions. Therefore, it is recommended that future studies include these control conditions, to demonstrate that the effects are due to the specific interventions. A limitation of this review is that the study samples reviewed were largely based on prison populations, while many offenders are treated and seen in outpatient care facilities or under probation supervision. It may be possible that prison populations have worse ABI-related impairments in comparison to forensic outpatients care populations, although this needs further investigation given the possible influence of other psychiatric disorders in the prison population. A second limitation is that the studies reviewed used different ABI assessment methods, namely self-report measurements, semi-structured interviews and information from medical records, with and without considering ABI severity. Other recommendations for future research could be to use a standardized ABI assessment instrument, such as a structured interview to asses both (a history of) ABI and current cognitive deficits. In clinical practices there are several instruments available that may be used in the forensic setting as well. For example, the Ohio State University (OSU) Traumatic Brain Injury (TBI) Identification Method (OSU TBI-ID) is a standardized procedure to elicit a person's lifetime history of TBI during a 3–5 min structured interview (60). Similar instruments, where necessary complemented with neuropsychological tests, would be a valuable addition to current practices in forensic settings. Longer follow-up periods, to study the long-term outcomes of the intervention, are also warranted. Ideally a follow-up of 2 year after release is incorporated, since most recidivism occurs within that period (61). Finally, larger sample sizes and including recidivism as an outcome measure are important suggestions for improving future research. Almost all research on ABI in the offender population focused on TBI and virtually none on non-traumatic brain injury. This might be because TBI is more common in offender populations than non-traumatic brain injury, because of a lack of focus in assessment non-traumatic brain injury, or because of limited knowledge of non-traumatic brain injury in forensic settings. Further suggestions for interventions to address ABI related impairments and behavioral changes in offender populations can be derived from literature on neuropsychological rehabilitation and treatment after ABI in the general, i.e., non-offender, populations. However, one difficulty when comparing ABI-related interventions from the general population with ABI interventions for forensic settings, is that the former does not focus on reducing recidivism, which is an important outcome measurement in the latter.

## Conclusions

The aim of the current study was to review the literature on interventions and treatment in offender populations suffering from ABI, or the influence of ABI on treatment. In the long run, improved treatment options and treatment outcomes may reduce the ABI-related impairments in offenders and, ultimately reduce recidivism. A systematic literature search identified a limited number of intervention studies in the offender population (*N* = 4) that reported some positive effects on interpersonal behavior, cognition and recidivism. However, due to methodological limitations the findings may not be generalizable to other samples and interpretations of intervention effectiveness should be considered with caution. Future studies are warranted, since this does seem as an important venue. Suggestions for future studies include standardized assessment of ABI, longer follow-up periods and inclusion of recidivism as outcome measure.

## Author Contributions

EdG, SN, MM, and FJ developed the research question. EdG carried out the database search. JH replicated the database search. EdG, MM, and SN drafted the manuscript. Earlier versions of the manuscript were revised by MM, SN, and JvH, more finalized versions were revised by MM, SN, JvH, FJ, JH, CG, TF, and FK. Preparation of the final manuscript in line with journal guidelines and submission were done by EdG, MM, and SN. All authors contributed to the article and approved the submitted version.

## Conflict of Interest

The authors declare that the research was conducted in the absence of any commercial or financial relationships that could be construed as a potential conflict of interest.
